# Icy Intrigue: A Case Report of an Unusual Encounter With an Ice Pick Injury

**DOI:** 10.7759/cureus.40805

**Published:** 2023-06-22

**Authors:** Yashpal S, Vikas Vaibhav, Salu Chandran, Lakhan Lal Navlani, Ashish R Bhute, Raviprakash Meshram

**Affiliations:** 1 Forensic Medicine and Toxicology, All India Institute of Medical Sciences, Rishikesh, Rishikesh, IND

**Keywords:** ice pick suicides, forensic implication of ice pick wounds, penetrating injury, ice pick assault, ice pick wounds

## Abstract

An ice pick is rarely observed as a weapon of offense in homicide or suicide. The severity of injuries produced by an ice pick ranges from circular contused abrasions to punctured wounds with clean-cut margins. We present a case of a self-inflicted ice pick injury to the chest autopsied at the AIIMS Rishikesh morgue. During this study, we noticed and analyzed the unusual presentation of injury by an ice pick, contrary to the injuries generally observed in ice pick cases. We highlight a peculiar case of a homicidal ice pick injury observed during the autopsy. The presentation deviated from typical patterns, with the weapon penetrating the victim’s body in an unconventional manner. The findings challenge traditional assumptions regarding ice pick injuries and underscore the importance of comprehensive forensic analysis. Understanding and documenting atypical presentations of such injuries can aid forensic experts in accurately determining the cause and manner of death, facilitating the pursuit of justice in criminal investigations.

## Introduction

Wounds to the chest can be produced by both blunt and sharp weapons or force. Injuries due to sharp weapons can be classified into incised wounds and stab wounds. Stab or punctured wounds are further classified as perforating and penetrating injuries. Penetrating injuries have a track that terminates inside the body while perforating injuries are through and through puncture wounds [1]. Weapons that may inflict penetrating and perforating injuries should have a pointed end, like knives, screwdrivers, scissors, or ice picks. Injuries due to ice picks are rare nowadays due to their declining use as a regular household instrument [2]. This case report is about an unusual presentation of self-inflicted, fatal ice-pick injury to the chest in a 30-year-old male.

## Case presentation

**Figure 1:**
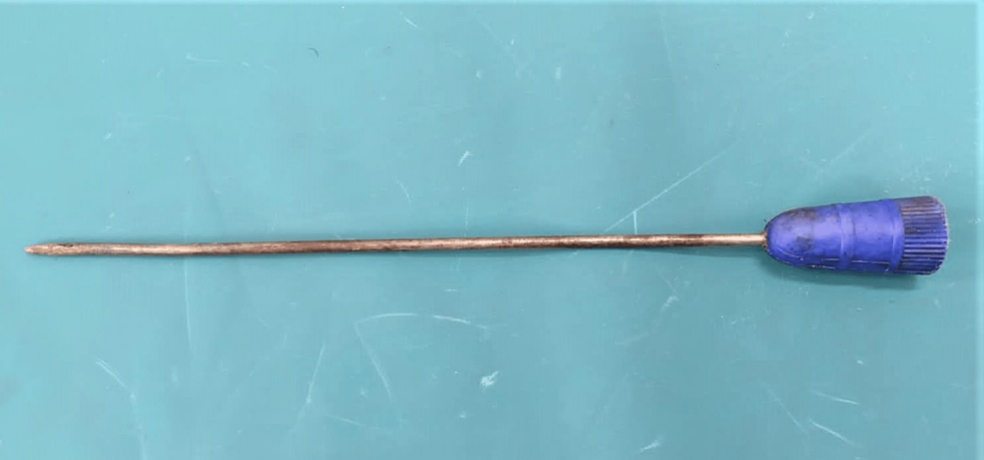
Weapon (ice pick) used by the victim

The patient was brought to AIIMS Rishikesh emergency in an unresponsive state with an alleged history of self-inflicted injury over the front of the neck and left side of the chest by an ice pick at his home (Figure 1). Primary treatment (suturing) and removal of the weapon were done at a nearby hospital. Later, he was referred to AIIMS Rishikesh where he presented in the gasping stage two hours after the incident. He was given CPR as per the advanced cardiac life support (ACLS) protocol, but despite best efforts, the patient could not survive and was declared brought dead. A post-mortem examination was conducted after 34 hours of death. As per the history given by relatives, the incident was witnessed by his mother. On further inquiry, the family was informed about a history of multiple substance abuse by the deceased.

Autopsy findings

There were multiple injuries present over the body; a brief description of external and internal injuries as described below.

External Injuries

Several external injuries were present over the neck viz. multiple, horizontal, and parallel linear scratch abrasions were present over the middle part of the front of the neck at the level of thyroid cartilage with size varying from 9.5 cm x 0.1 cm to 8.4 cm x 0.1 cm (suggestive of hesitation cuts). A puncture wound of size 2.1 cm x 0.5 cm x tracheal cavity deep was present over the front of the neck in the midline situated 9.3 cm above the sternal notch. The margins of the wound were clean-cut.

**Figure 2:**
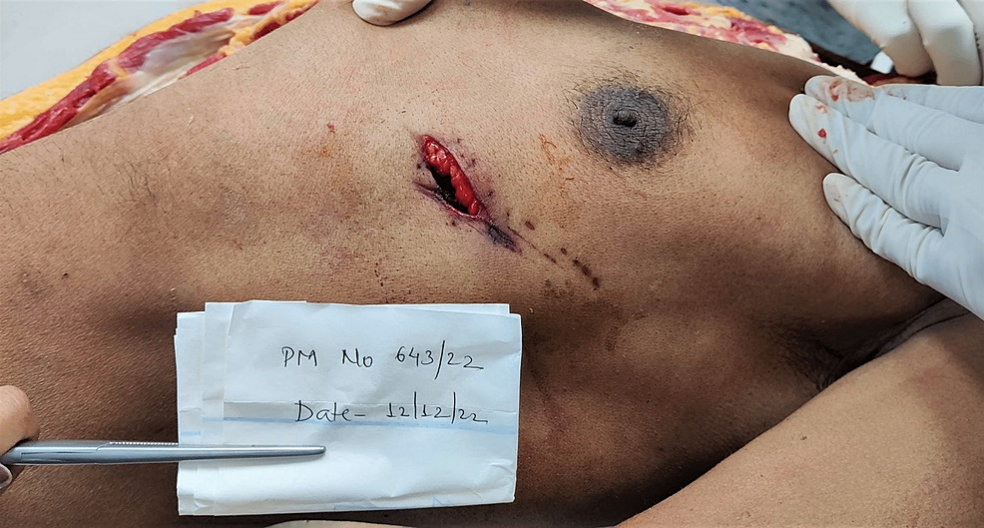
Wound over the chest

**Figure 3:**
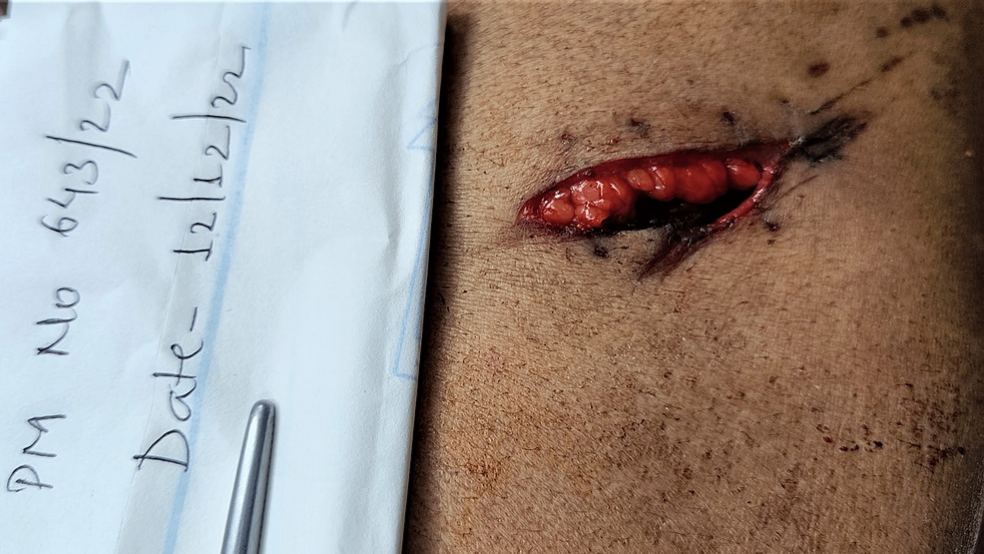
Wound over the chest

Injuries present over the chest were: a sutured wound of length 3.5 cm with four sutures was present over the left side of the chest, parallel to the plane of ribs and corresponding to the fifth intercostal space, the upper, lateral end of the wound situated 7.5 cm lateral to the midline, and 4.2 cm below the left nipple. On removal of sutures, a spindle-shaped wound of size 3.5 cm x 1.5 cm x thoracic cavity deep was present with subcutaneous fat protruding from the medial edge with irregular margins. The medial lower end was acute and the upper lateral end was blunt. Scratch abrasions were present mimicking tailing from medial to lateral and from downward to above as shown in Figures 2, 3. This was not consistent with the usual tailing produced by a knife. There was palpable crepitation in subcutaneous tissue suggestive of subcutaneous emphysema, with extravasation of blood under the surface of the wound (Figures 2, 3).

**Figure 4:**
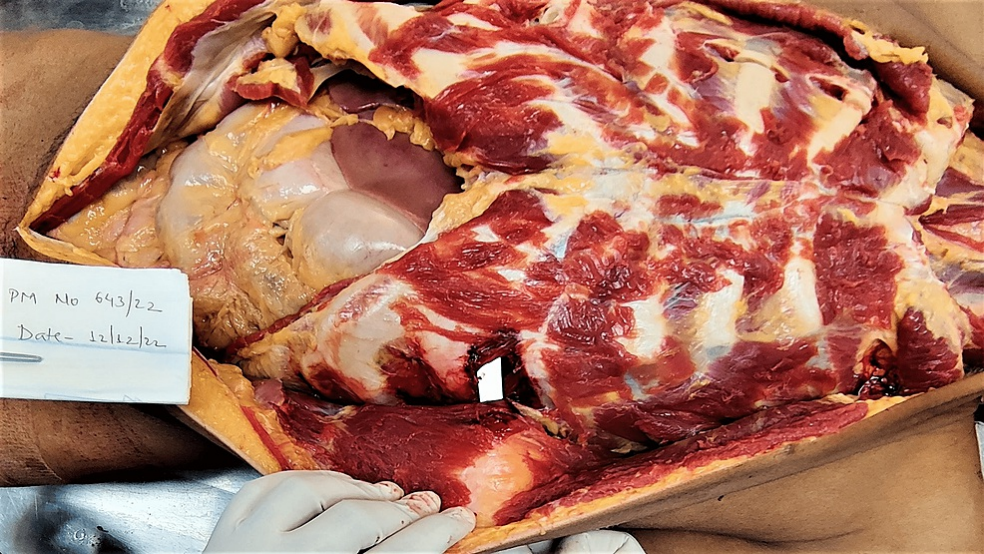
Tract of the wound

**Figure 5:**
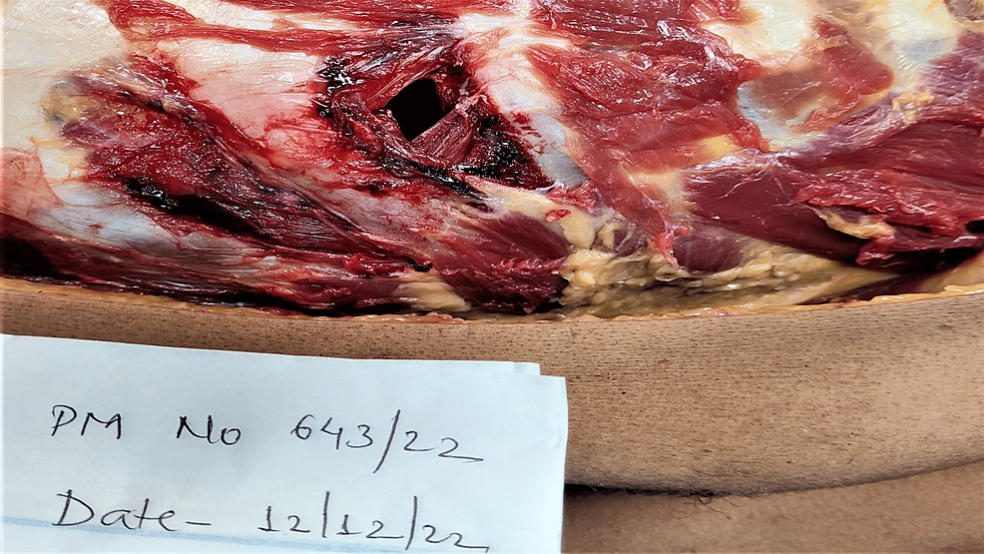
Tract of the wound

The tract of wound present over the chest was starting from the skin, as described above, piercing the subcutaneous tissue, pectoralis major muscle, and fifth intercostal space along the upper border of the fifth rib through the intercostal muscle creating a defect of size 1.5 cm x 1.4 cm. The track further extended into the pericardial sac producing a defect of size 2.1 cm x 0.4 cm. Further, the tract of the wound pierced the left ventricular wall over the diaphragmatic surface (inferior surface) of the heart; terminated by producing a contusion over the anterior papillary muscle in the left ventricular cavity. The direction of the wound is from left to right, downward to upward, and front to back (Figures 4, 5).

Several other injuries present were a reddish contused abrasion of length 0.9 cm x 0.1 cm present over the left side of the chest extending from the lower border of injury no. 2 situated 5.1 cm below the left nipple and 9.2 cm lateral to the midline. A reddish contused abrasion of size 1.9 cm x 0.5 cm was present over the left side of the chest extending from the upper lateral end of injury no. 2 and 3.8 cm below the left nipple. A reddish contused abrasion of size 5.9 cm x 0.1 cm was present over the left side of the chest situated 0.3 cm above injury no. 4, and multiple reddish contused abrasions of sizes varying from 0.3 cm x 0.2 cm to 0.1 cm x 0.1 cm were present over the left side of chest situated 0.3 cm above injury no.5.

Internal Injuries

**Figure 6:**
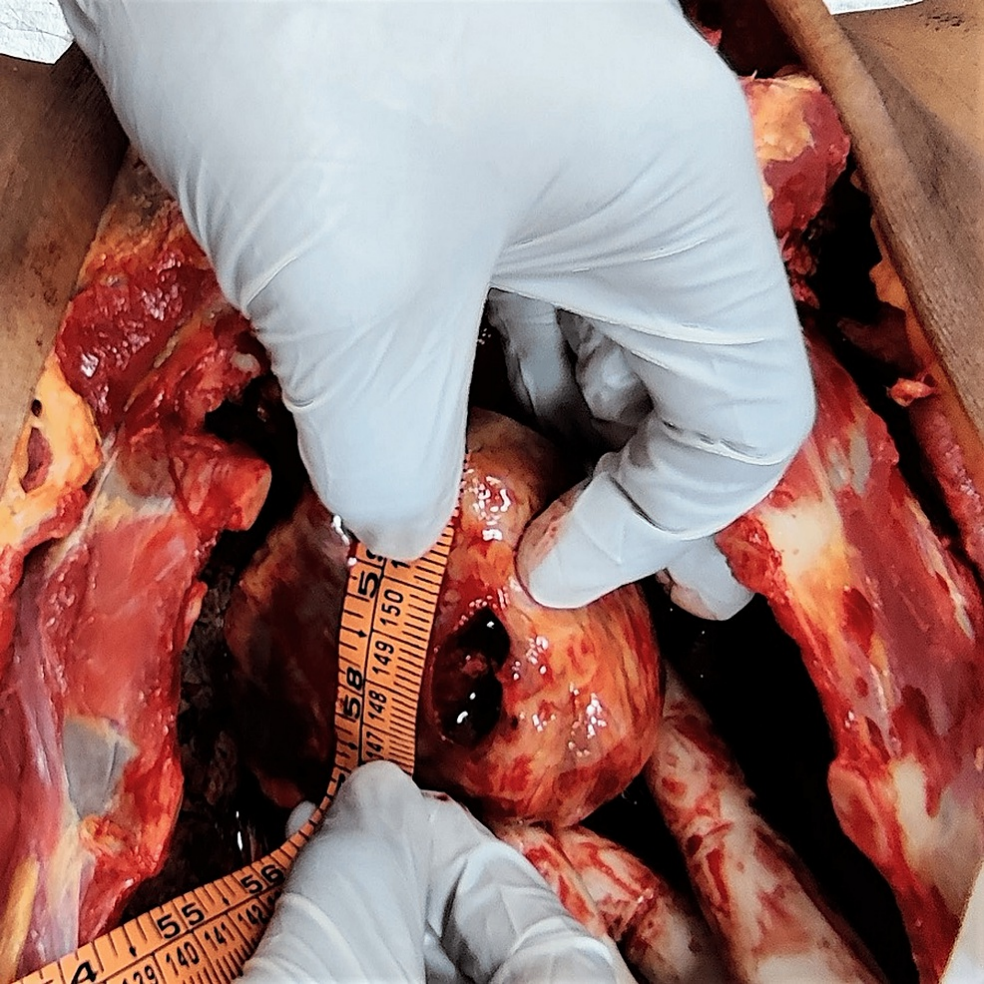
Defect over the inferior surface of the heart

**Figure 7:**
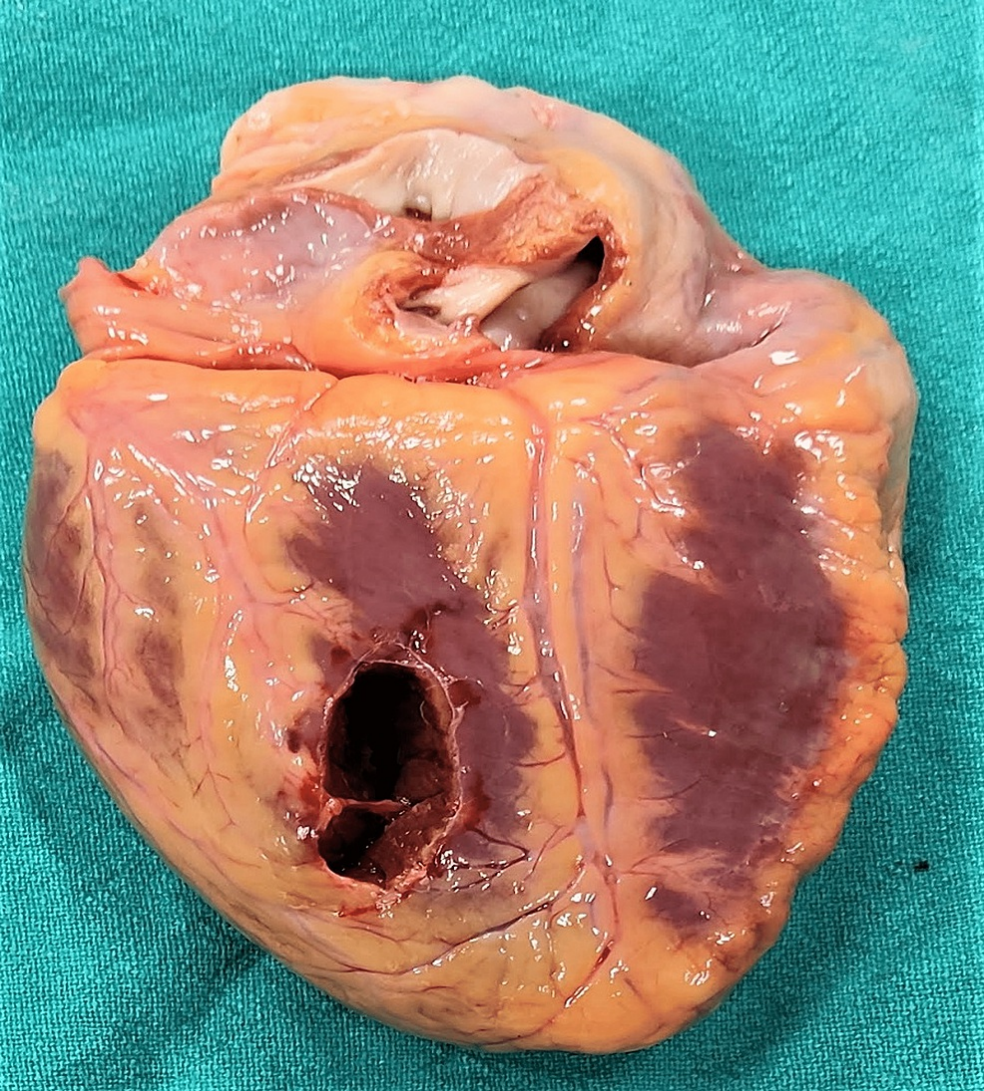
Defect over the inferior surface of the heart with tissue bridging

Injuries corresponding to the external injuries were: a defect of size 0.5cm x 0.3cm present over the superior junction of thyroid laminas in the midline. There was also extravasation of blood present over the strap muscles of the neck, namely, sternohyoid, thyrohyoid, and sternothyroid. A circular perforating wound of size 0.3 cm x 0.3 cm passing through the parenchyma was present over the costal and medial surfaces in the lower part of the left upper lobe of the left lung. A defect of size 0.2 cm x 0.1 cm was present over the pericardium and a defect of size 3.9 cm x 1.1 cm x cavity deep was present over the inferior surface of the left ventricle with irregular margins and tissue bridging. On the cut section, a contusion of size 0.3x0.2 cm was present over the papillary muscle of the anterior wall of the left ventricle (Figures 6, 7).

## Discussion

**Figure 8:**
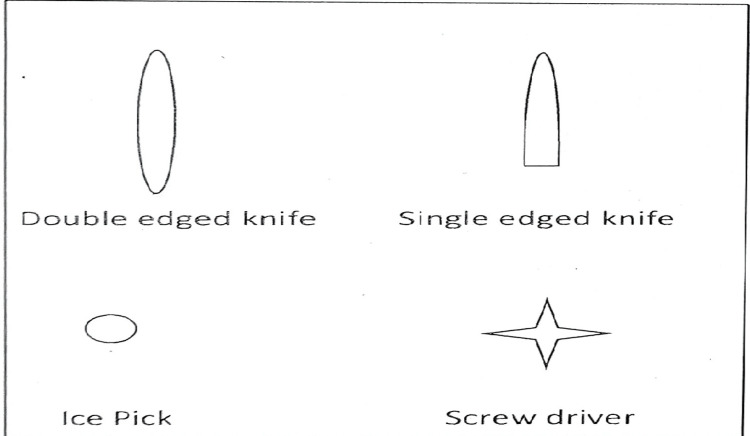
Common shapes of penetrating injuries

Stab injuries to the chest by ice pick are more commonly homicidal rather than accidental or suicidal [3]. In homicides, fatal injury is associated with multiple non-fatal injuries over the part of the body encountering the weapon during the scuffle. Suicidal injuries are often accompanied by tentative injuries over accessible parts of the body like the wrist and forearm [4]. Hesitation cuts may be present as old scars or fresh injuries produced before a fatal wound [5]. Fatal injuries in suicidal cases are generally present over sites of predilection such as the neck, chest, and wrist [5]. There can be different shapes of penetrating injuries depending upon the weapon of offense (Figure 8).

On initial inspection, the injury over the chest appeared to be a lacerated wound with the presence of subcutaneous fat protruding into the defect; the irregular margins and unusual tailing present suggested that they could not be due to a stab by a knife. On examination with a hand lens, the tailing was contused abrasions. These abrasions were suggestive of hesitation or tentative injuries. On further dissection of the tract of the injury, the pectoralis muscle had a rhomboid defect with irregularly cut muscle fibers at the margins. The lower lobe of the left lung (lingula) showed a circular perforating wound, the pericardium showed circular tears, and a penetrating wound over the left ventricle and a contusion were present over the papillary muscle of the left ventricle.

Taking into account the history, presentation of injury, and autopsy findings, the injury did not appear to be caused by a knife or an ice pick as the usual dimensions of ice picks available on the market did not match the size of the injury and the pattern of injury. Injuries inflicted by an ice pick are usually circular contusions, abrasion, or puncture wounds; however, a pattern of injury can vary significantly as observed in our case. So, this dilemma was sorted out by following observations.

The basis of analysis of injuries present in our case is based on the history given by the investigating officer, relatives, circumstantial evidence, and analysis of various mechanisms causing the unusual presentation of ice pick injury. It was observed that in injury no. 1, both a puncture wound and abrasions were present (might be hesitation cuts) possibly due to the pointed end of the ice pick or knife. Injury no. 2 showed the presence of subcutaneous fat, giving the appearance of a stab wound by a single-edged knife, but the history and weapon recovered were strong circumstantial evidence of the injury being inflicted by an ice pick rather than a knife. This unusual and peculiar presentation of the injury can be explained by the following theories:

One of the theories is the Kennedy phenomenon, i.e. suturing [6]. Another theory is due to the relative movement between the ice pick and the body, which may be due to the trial of pulling back by the person himself or someone else, or while transporting the injured person to the hospital where it was removed from the chest, or it can be due to puncturing of the skin multiple times by the person.

Injury no. 3 could be due to the oblique direction of stabbing or due to pulling out of the ice pick. All the other injuries, viz. injuries nos. 4, 5, and 6, are due to unsuccessful attempts of stabbing prior to the fatal stab wound inflicted to the chest. On internal examination, it was observed that there was injury over the pectoralis, which was a rhomboid defect with irregularly cut muscle fibers over the margins. The injury over the heart was with incomplete intervening bridging of tissue, suggesting relative movement of the ice pick and body or stabbing twice [7].

There are numerous factors, such as unemployment, substance use disorder, psychiatric illness, or unstable relationships, leading to suicidal ideations [8]. Two of the aforesaid reasons present in our case were unemployment and substance use.

## Conclusions

A forensic pathologist should be vigilant and well-versed with factors such as the elasticity of the skin, the part of the body over which injury is inflicted, and movement of the body or weapon inside the body that can cause unusual variations to the injuries. In the presented case, several factors were in favor of an ice pick as a weapon of offense, viz. the irregular margin with tissue bridging, the circular perforating wound of the lung with margins containing multiple semi-circular indentations, and contusion over the papillary muscle. The deviation from conventional patterns underscores the need for comprehensive and open-minded analysis in forensic investigations. By recognizing and documenting such unusual presentations, forensic experts can refine their understanding of weapon-related injuries, leading to more accurate determinations of cause and manner of death. This case highlights the dynamic nature of forensic medicine and emphasizes the importance of continuous learning and adaptation in the pursuit of justice.

## References

[1] Narayan Reddy KS (2022). Stab wound or punctured wound. Essentials of Forensic Medicine and Toxicology.

[2] DiMaio VJ, DiMaio D (2001). Blunt trauma wound. Forensic Pathology.

[3] Krishna K, Behera C, Singh SR, Bhardwaj DN (2013). Ice pick death: a case report and discussion of the injury pattern. J Forensic Leg Med.

[4] Knight B (1975). The dynamics of stab wounds. Forensic Sci.

[5] Bernard K, Pekka S (2016). Self-inflicted injuries, suicidal knife wounds. Knight’s Forensic Pathology.

[6] Nalli NR (2018). Gunshot-wound dynamics model for John F. Kennedy assassination. Heliyon.

[7] Bleetman A, Watson CH, Horsfall I, Champion SM (2003). Wounding patterns and human performance in knife attacks: optimising the protection provided by knife-resistant body armour. J Clin Forensic Med.

[8] Mathieu S, Treloar A, Hawgood J, Ross V, Kõlves K (2022). The role of unemployment, financial hardship, and economic recession on suicidal behaviors and interventions to mitigate their impact: a review. Front Public Health.

